# Rising Cesarean Section Rates in Pakistan: Analyzing Frequency and Key Risk Factors in a Tertiary-Care Hospital

**DOI:** 10.7759/cureus.72333

**Published:** 2024-10-24

**Authors:** Ismail Jadoon, Mudassir Khattak, Khoula S Mughal, Umair S Mughal, Majid Khan, Saud A Abdulsamad, Abdulghani A Naeem

**Affiliations:** 1 Pharmacy, COMSATS University Islamabad, Abbottabad, PAK; 2 Pharmacy, Abasyn University, Peshawar, PAK; 3 Medicine, Ayub Teaching Hospital, Abbottabad, PAK; 4 Basic Sciences, College of Science and Health Professions, King Saud bin Abdulaziz University for Health Sciences, Jeddah, SAU; 5 Pharmacology, King Abdullah International Medical Research Center, Jeddah, SAU

**Keywords:** cesarean section, determinants, elective, emergency, haemorrhage, perinatal mortality, pre-eclampsia

## Abstract

The increasing frequency of cesarean sections (CS) has emerged as a significant public health concern, particularly in Pakistan. This cross-sectional study aimed to assess the prevalence of CS and its contributing factors among mothers in an underdeveloped region of the country. Data were collected from 250 delivery cases using a self-developed questionnaire. Among the participants, 110 (44%) had undergone CS, with 67 (60.9%) being emergency procedures and 43 (39.1%) elective. Statistical analysis revealed that younger maternal age (p = 0.04), type of CS (p = 0.05), and education level (p = 0.04) were significantly associated with higher rates of CS. Major medical risk factors contributing to CS included preeclampsia/eclampsia, vaginal hemorrhage, gestational diabetes, and hypertension (p < 0.001). Furthermore, urban residence, younger maternal age (20-24 years), and preterm gestational age were also identified as significant contributors. The findings suggest that the high rate of CS (44%) in the study population is influenced not only by physiological and medical factors but also by demographic elements such as education, income, and type of delivery. To mitigate unnecessary CS, community-focused educational interventions targeting at-risk populations should be implemented during pregnancy. Effective measures are essential to reduce the rising CS rates in Pakistan.

## Introduction

Cesarean sections (CS) are a common surgical procedure used to save the lives of both the mother and the fetus and to prevent complications during childbirth [1]. An individual's decision regarding the delivery method is greatly influenced by how they perceive and cope with labor pain, how they comprehend what labor pain is, how they manage with pain, and other behaviors related to labor pain [2].

Globally, the incidence of CS varies greatly across developed and developing nations; rates in advanced countries range from 23.8% to 50%, whereas incidence in underdeveloped nations is typically less than 10% [3]. WHO reports indicate that CS rates are rising globally, accounting for 21% of all deliveries at present, and projected to rise to 29% by 2030 [4]. This is more than the WHO's suggested threshold of 15% or less for the ideal ratio of advantages to disadvantages [5]. A woman's chance of experiencing pregnancy-related morbidity and death is increased dramatically after a cesarean delivery, according to a report published by the American College of Obstetricians and Gynecologists (ACOG). According to the survey, women who have CS deliveries die at a rate of about 35.9 deaths per 100,000 live births or successful deliveries, whereas women who have vaginal deliveries die at a rate of 9.2 deaths per 100,000 live births [6].

There are 348 maternal deaths for every million babies born due to CS, with Pakistan accounting for 7% of the global incidence of fatalities [7]. Evidence of a growing tendency has been observed in South Asian nations, including Pakistan, where the proportion of CS deliveries increased from 3.2% in 1990 to 20% in 2018 [8]. Pakistan has achieved advances in several health metrics, but there are still big problems with maternal and child healthcare. Pakistan is among the top 10 countries in the world where 59% of maternal deaths worldwide are attributed to CS [9]. Despite a decline in the maternal mortality ratio (MMR) from 521 to 178 incidents per 100,000 people between 1990 and 2015, the country failed to achieve the 2015 target of 130 CS per 100,000 people [10].

To our knowledge, the Gynecology Ward of the Ayub Teaching Hospital in Abbottabad has not conducted a recent study using the same methodology. The current work was prompted by this research gap. With the enormous influence that CS have on maternal and newborn morbidity and death, this study aims to ascertain the frequency of CS as well as evaluate the different risk factors of their occurrence.

## Materials and methods

Study design and ethical approval

This observational cross-sectional research was conducted at Ayub Teaching Hospital, focusing on the gynecology outpatient departments and wards. Data collection took place over a three-month period, from February 2022 to March 2022. The study was conducted in conformity with the regulations, standards, and ethics established by the Institutional Ethics Review Committee at Abasyn University Peshawar (Ref. No. IERC-AUP 2024-021). The study was performed as per the declaration of Helsinki.

Study participants

The study population consisted of women that had given birth at the obstetrics and gynecology department of the hospital. The study involved pregnant female patients between the ages of 18 and 55, who provided informed consent to participate. The inclusion criteria were limited to pregnant women within this age range. 

Sample size determination and sampling technique

The sample size was calculated using a formula for estimating a proportion in a population, with a 5% margin of error and 95% confidence level, corresponding to a Z-value of 1.96. The calculated sample size was 240, which was increased to 250 to account for the 30% prevalence of CS deliveries and a 10% non-response rate. Participants were chosen through a convenience sampling method.

Study tool and data collection process

A self-developed questionnaire was designed for data collection. The questionnaire comprised two domains. The first domain focused on sociodemographic factors, including the age, caste, education, and place of residence of patients, as well as their occupation. It also covered the nature of cesarean sections and the socioeconomic status of patients, categorized as lower (monthly income < 25,000), middle (25,000-50,000), and upper-middle class (50,000-100,000) in Pakistani rupees. The second domain comprised questions regarding pregnancy complications. Data were gathered from participants via structured interviews and reviews of their medical records. Demographic details, such as age and residence, were recorded, along with medical histories, including hypertension and pregnancy status, for the pregnant participants.

Data analysis

Descriptive statistics, such as frequencies and percentages, were employed to analyze the data. Comparative analyses were conducted using the chi-square test to assess differences between normal deliveries and CS. Fisher’s exact test was applied when cell counts were below 5. The statistical analysis was performed using SPSS software (version 25).

## Results

**Figure 1:**
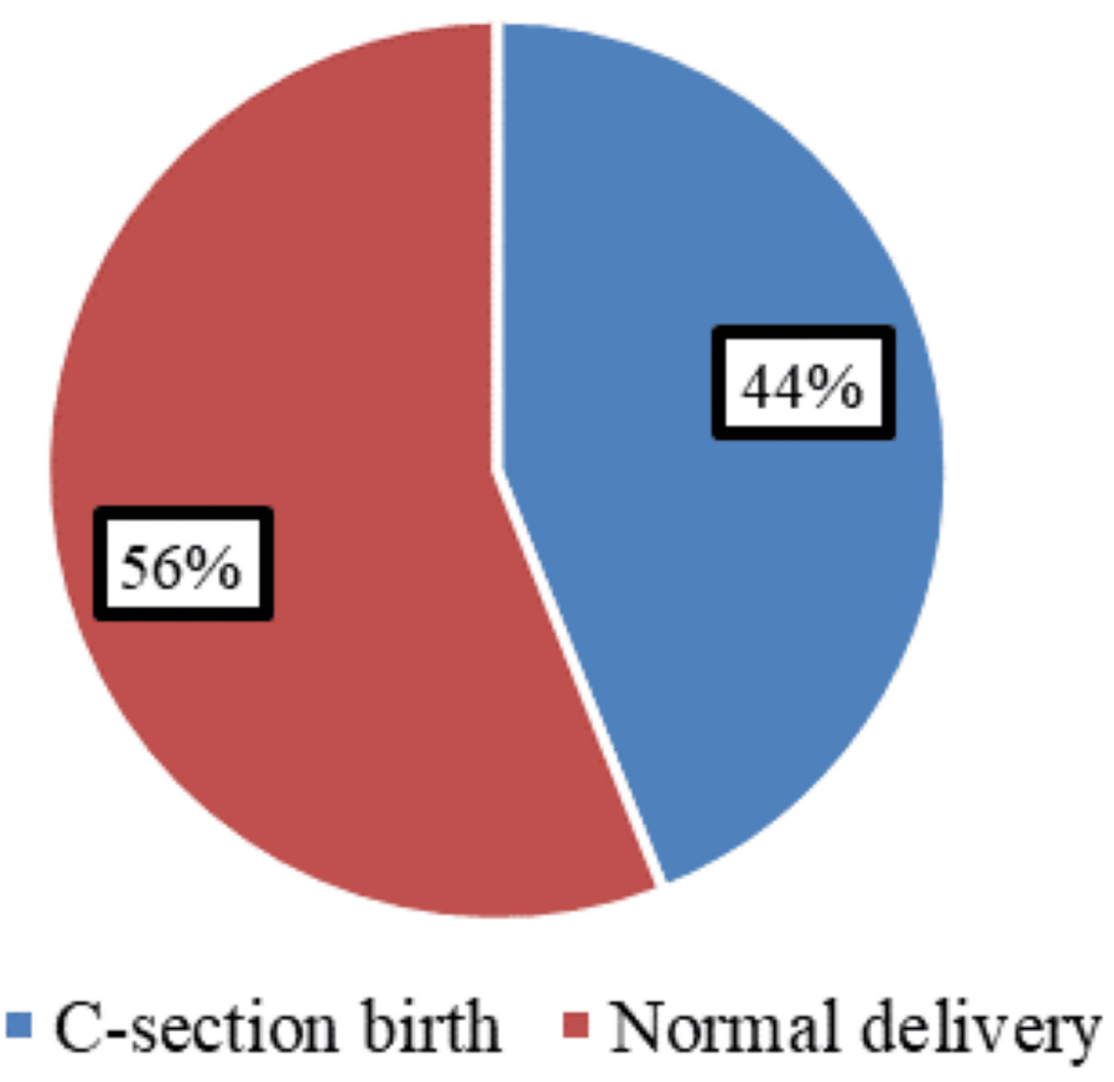
Rate of CS in the study population CS: Cesarean section

A total of 250 women were recruited for the study, with a mean age of 27.5 ± 6.0 years. Of these, 110 women (44%) underwent CS, while 140 (56%) had normal deliveries (Figure 1). 

Among the cesarean cases, 43 (39.1%) were elective CS, while 67 (60.9%) were emergency CS. Table 1 outlines the sociodemographic and obstetric characteristics of the participants. The maternal age of women who had CS ranged from 18 to 50 years. The age group distribution revealed that most women undergoing CS were in the 20-29-year age group, with 67 (77%) in the elective group and 48 (79%) in the emergency group. In comparison, only 14 (12.7%) women were in the 30-34 age group, including 8 (9%) who had elective and 6 (9%) who had emergency CS. Additionally, 9% of women in both groups were aged 19 years. However, the age distribution between elective and emergency CS groups did not show a significant difference (p = 0.06).

**Table 1:** Sample characteristics of the study (N=250) Fisher exact test was applied when the cell counts were less than 5 and for others Chi-squared exact tests for categorical variables. *p < 0.05 CS: Cesarean section

Characteristics	Total = 250 (N (%))	Cesarean Birth (N (%))	Vaginal Birth (N (%))	p-value
Age (years)				
17-19	11 (4.4)	8 (72.7)	3 (27.2)	
20-24	76 (30.4)	35 (46.05)	42 (53.95)	
25-29	71 (28.4)	29 (40.8)	41 (59.2)	0.06
29-34	68 (27.2)	27 (39.7)	41 (60.3)	
>35	24 (9.6)	10 (41.6)	14 (58.4)	
Caste of Patients				
Pathan	73 (29.2)	35 (47.9)	39 (52.1)	
Awan	43 (17.2)	20 (46.5)	23 (53.5)	
Gilgiti	5 (2)	1 (20)	4 (80)	
Kohistani	7 (2.8)	2 (28.5)	5 (71.5)	0.23
Tanoli	21 (8.4)	8 (38)	13 (62)	
Syed	20 (8)	6 (30)	14 (70)	
Others	80 (32)	38 (47.5)	42 (52.5)	
Residence				
Urban	91 (36.4)	38 (41.7)	53 (58.3)	0.75
Rural	159 (63.6)	72 (45.2)	87 (54.5)	
Socioeconomic status of patients				
Poor (< Rs 25,000)	33 (13.2)	10 (30.3)	23 (59.7)	
Middle (Rs 25,000-50,000)	204 (81.6)	96 (47)	108 (53)	0.02*
Upper Middle (Rs 50,000-100,000)	13 (5.2)	4 (30.7)	9 (59.3)	
Level of education of patients				
Uneducated	88 (35.2)	49 (55.6)	39 (44.4)	
Primary (1-6)	49 (19.6)	18 (36.7)	31 (63.3)	
Secondary (7-12)	51 (20.4)	18 (35.2)	33 (64.8)	0.04*
Tertiary (bachelors)	42 (16.8)	19 (45.2)	23 (54.8)	
Higher (masters)	20 (8)	8 (40)	12 (60)	
Occupation of patients				
Unemployed	213 (85.2)	104 (48.8)	109 (51.2)	0.09
Employed	37 (14.8)	6 (16.2)	31 (83.8)	
Nature of CS				
Emergency	67 (60.9)	67 (60.9)		0.05*
Elective	43 (39.1)	43 (39.1)		

The Pathan ethnic group had the highest representation, with 74 participants (29.6%), of whom 35 (47.2%) underwent CS (Table 1). The majority of participants 204 (81.6%) belonged to the middle socioeconomic class. Economic status of the individuals have a significant association with the CS with p= 0.02, and of these, 96 (47%) had CS. Rural residents comprised the larger portion, with 159 women (63.6%) from rural areas, 72 (45.2%) of whom had CS, compared to 91 women (36.4%) from urban areas, with 38 (41.7%) undergoing CS. Literacy level was significantly associated with the CS performed (p = 0.04). Among CS patients, 104 (94.5%) were unemployed, while only 6 (5.5%) were employed (p = 0.09).

The various indications for CS were examined in this study. The most common reason was a previous CS, accounting for 53 cases (48.2%). Other notable indications included fetal distress in 18 cases (16.3%), low fetal weight in 5 cases (4.5%), fetal bradycardia in 3 cases (2.7%), fetal tachycardia in 4 cases (3.6%), premature rupture of membranes in 4 cases (3.6%), delayed labor in 6 cases (5.5%), a history of obstructed labor in 4 cases (3.6%), and chronic hypertension in 19 cases (17.3%). These findings are illustrated in Table 2.

**Table 2:** Determinants of CS CS: Cesarean section

Variables	Frequency (n)	Percentage (%)
Previous CS	53	48.2
Hypertensive disorder	19	17.3
Fetal distress	18	16.3
Delayed labor	6	5.5
Low fetal weight	5	4.5
Fetal tachycardia	4	3.6
Fetal bradycardia	3	2.7
History of obstructed labor	4	3.6
Premature rupture of membrane	4	3.6

Of the 110 C-section cases, a significant number experienced pregnancy-related complications. The most common complications included gestational diabetes (13 out of 25 total cases), vaginal hemorrhage (13 out of 13), and hypertension (11 out of 19 cases). Major risk factors for CS included pregnancy complications such as gestational diabetes, hypertension, preeclampsia/eclampsia, and vaginal hemorrhage, all of which showed a significant association with CS (p < 0.001). Table 3 provides an overview of the pregnancy-related complications.

**Table 3:** The association of pregnancy complications with the mode of delivery Fisher exact test was applied. *p < 0.05 indicates significance.

Variables	Total = 250 (N (%))	Cesarean Birth (N (%))	Vaginal Birth (N (%))	p-value
No pregnancy complications	168 (67.2)	73 (43.4)	95 (56.5)	
Gestational diabetes	25 (10)	13 (52)	12 (48)	
Hypertension	19 (7.6)	11 (57.8)	8 (42.2)	<0.001
Pre-eclampsia/eclampsia	14 (5.6)	4 (28.5)	10 (71.5)	
Vaginal bleeding	13 (5.2)	13 (100)	0 (0)	
Others	11 (4.4)	9 (81.8)	2 (18.2)	

## Discussion

The present study was conducted at Ayub Teaching Hospital, Abbottabad, to assess the frequency of CS in the Hazara division and to explore associated risk factors. The findings indicated that multiple elements contribute to the CS rate. Among the total of 250 deliveries during the study period, there were 110 cases of CS, accounting for 44%. This rate is notably higher than in previous studies although some studies reported even higher rates, with figures of 69.7% and 48.98%, respectively [11,12].

In our study, the majority of women (51%) in the elective CS group and 30% in the emergency group were aged 20-24 years. This contrasts with findings from Verma et al., where most women were in the 26-30 age group, with 51% in the elective and 49% in the emergency category [13]. The age of women undergoing CS in our study ranged from 18 to 50 years, with 77% of those in the elective group and 79% in the emergency group falling within the 20-29 age range. Only 12.7% were in the 30-34 age group, and very few were aged 19. In contrast, Janoudi et al. reported that C-section rates increased with age, with 26.2% for ages 20-34, 35.9% for ages 35-40, and 43.1% for those over 40 [14]. The higher prevalence of CS among younger women in our study may be linked to the cultural context of early marriage prevalent in Pakistan, particularly in Khyber Pakhtunkhwa.

Additionally, we found that CS rates were higher among rural women (65.5%) compared to urban women (34.5%), likely due to greater patient traffic from rural areas. This trend suggests that factors such as lower literacy rates and economic status contribute to the preference for CS. Specifically, our socioeconomic analysis indicated that only 9.1% of women were categorized as poor, while 87.3% were from lower-middle-class backgrounds, and 3.6% from upper-middle-class backgrounds. This is contrary to the result of the study by Rasool et al., who reported higher CS rates among urban women due to reduced physical activity [5]. Other studies have also identified significant associations between CS likelihood and factors such as area of residence, educational attainment, and wealth index, reinforcing findings from developing countries [1].

Among the 110 women in our study, a substantial majority (94.5%) were housewives, with only a small percentage in government or private employment, potentially affecting their physical activity levels [15,16]. Interestingly, women from the Pathan ethnic group exhibited the highest rate of CS (31.8%), consistent with previous research in Khyber Pakhtunkhwa [17-20].

In our study, 53 women (48.2%) had a history of previous CS, while 57 (51.8%) had no prior CS. Among the total cases, 43 (39.1%) underwent elective CS and 67 (60.9%) had emergency procedures. This aligns with findings from another study, which reported a slightly higher rate of emergency CS at 81.95% [21]. The unpredictable nature of emergency CS often leads some women to prefer the option of scheduling their births. If obstetricians could guarantee a successful vaginal delivery, it is likely that fewer women would choose cesarean delivery. Research indicates that only a small percentage of women who deliver vaginally for the first time later request a CS, usually due to specific issues from their initial experience, such as inadequate pain management, prolonged labor, or significant tearing [22,23].

Currently, there is no evidence to suggest that elective CS are safer than vaginal deliveries; in fact, most studies indicate that CS carry greater risks [24]. Consequently, it is essential for healthcare providers to advocate for vaginal delivery as the preferred method. Additionally, various pregnancy complications contribute to the rising rates of cesarean deliveries [25]. In our findings, the most common factors leading to CS included a previous cesarean (48.2%), hypertensive disorders (17.3%), and fetal distress (16.3%), with other factors such as delayed labor (5.5%) and low fetal weight (4.5%) also noted. These results are consistent with several studies conducted in low- and middle-income countries [26-30]. Globally, fetal distress has a prevalence of around 20% and accounted for approximately 16% of C-sections in tertiary hospitals in Bangladesh [27,28]. Other contributing factors include fetal distress, lack of labor progression, prior CS, hypertension, miscarriage, and stillbirth. Addressing these complications can be enhanced through improved maternal care, which includes easier access to healthcare facilities, regular follow-ups, and adherence to prescribed treatments during pregnancy. Providing women with a realistic clinical overview of CS may influence their decision regarding the mode of delivery.

In this study, several measures were taken to alleviate potential limitations. The current study had a small sample size, limiting the generalizability of the findings to the broader population. Additionally, the questionnaire primarily focused on evaluating the frequency of CS and its associated risk factors, which may not have captured all relevant aspects related to CS.

## Conclusions

Several factors contribute to the rising trend of CS among Pakistani women. Key contributors include previous CS, hypertension, fetal distress, and maternal age, particularly for emergency CS. This increasing trend places a burden on the healthcare system and strains families, potentially affecting maternal and child health. Some factors, such as maternal preferences, can be modified through counseling and education. Regular antenatal care visits, especially for monitoring blood pressure, can help reduce some risks. This study underscores the importance of educational interventions as a strategy to address the rising CS rates. Since this study was conducted in a single center, future research should include multiple hospitals to gain a broader understanding of the factors involved. Raising public awareness about these trends is also crucial.
